# Systematic Comparison of the Performances of *De Novo* Genome Assemblers for Oxford Nanopore Technology Reads From Piroplasm

**DOI:** 10.3389/fcimb.2021.696669

**Published:** 2021-08-16

**Authors:** Jinming Wang, Kai Chen, Qiaoyun Ren, Ying Zhang, Junlong Liu, Guangying Wang, Aihong Liu, Youquan Li, Guangyuan Liu, Jianxun Luo, Wei Miao, Jie Xiong, Hong Yin, Guiquan Guan

**Affiliations:** ^1^State Key Laboratory of Veterinary Etiological Biology, Key Laboratory of Veterinary Parasitology of Gansu Province, Lanzhou Veterinary Research Institute, Chinese Academy of Agricultural Science, Lanzhou, China; ^2^Key Laboratory of Aquatic Biodiversity and Conservation, Institute of Hydrobiology, Chinese Academy of Sciences, Wuhan, China; ^3^Key Laboratory of Functional Genomics and Molecular Diagnosis, Lanzhou Baiyuan Gene Technology Co., Ltd, Lanzhou, China; ^4^Jiangsu Co-Innovation Center for the Prevention and Control of Important Animal Infectious Disease and Zoonoses, Yangzhou University, Yangzhou, China

**Keywords:** *de novo* genome assembly, Oxford Nanopore Technology, long reads, *Babesia motasi*, Piroplasm

## Abstract

**Background:**

Emerging long reads sequencing technology has greatly changed the landscape of whole-genome sequencing, enabling scientists to contribute to decoding the genetic information of non-model species. The sequences generated by PacBio or Oxford Nanopore Technology (ONT) be assembled *de novo* before further analyses. Some genome *de novo* assemblers have been developed to assemble long reads generated by ONT. The performance of these assemblers has not been completely investigated. However, genome assembly is still a challenging task.

**Methods and Results:**

We systematically evaluated the performance of nine *de novo* assemblers for ONT on different coverage depth datasets. Several metrics were measured to determine the performance of these tools, including N50 length, sequence coverage, runtime, easy operation, accuracy of genome and genomic completeness in varying depths of coverage. Based on the results of our assessments, the performances of these tools are summarized as follows: 1) Coverage depth has a significant effect on genome quality; 2) The level of contiguity of the assembled genome varies dramatically among different *de novo* tools; 3) The correctness of an assembled genome is closely related to the completeness of the genome. More than 30× nanopore data can be assembled into a relatively complete genome, the quality of which is highly dependent on the polishing using next generation sequencing data.

**Conclusion:**

Considering the results of our investigation, the advantage and disadvantage of each tool are summarized and guidelines of selecting assembly tools are provided under specific conditions.

## Introduction

During the past two decades, the fast development of sequencing technology and rapid reduction in sequencing cost have enabled scientists to initiate projects to sequence the whole genome of any species. Next-generation sequencing (NGS) technology is relatively time saving, less labor intensive and more cost efficient (Lander et al., 2001). In particular, the dramatic success of the human genome project and the completeness of the whole-genome shotgun sequencing of some model organisms has inspired scientists to decode the genetic information of other non-model organisms (Abecasis et al., 2012). In most of these organisms, short reads from NGS were used, ranging from 35–150 bp paired reads and covering a depth range from 50 to 100-fold, which are too short to assemble genome-containing repetitive regions (Salzberg et al., 2012). To some extent, preparing the paired-end and mate-pair libraries and increasing the depth of sequencing coverage facilitate improvements in the accuracy and completeness of genomes. To obtain a complete genome, it is always necessary to make great additional efforts, such as Sanger sequencing and tailored assembly approaches (Salzberg et al., 2012). These methods cannot overcome the drawback of short reads especially in contiguity and assembling a genome with a high degree of repeats.

Thanks to the third generation sequencing technologies that are able to produce long reads, the issue of genomes containing highly repetitive regions has been overcome. It was first developed to produce long reads by Pacific Biosciences (PacBio) with a relatively high error rate (~10 to 15%) (Nagarajan and Pop, 2013). Subsequently, ONT was developed and provided reads of up to a few hundred thousand base pairs with tiny sequencers (Ashton et al., 2015; Laver et al., 2015). A 1D read from these sequencers has a ~75% rate of raw base accuracy, which has been improved to 80–88% for 2D reads (Ip et al., 2015; Lu et al., 2016). Assembly tools developed for NGS are not suitable for handling such long and high-error reads (Nagarajan and Pop, 2013). This intricate problem inspired scientists to develop new assembly and alignment algorithms, which were capable of making read error corrections using self-correction of PacBio reads/ONT reads or hybrid correction with NGS data.

One of the difficulties in assembling genomes comes from the newly emerged and existing many good assemblers, such as NECAT (Chen et al., 2021), Canu (Koren et al., 2017), wtdbg2 (Ruan and Li, 2020), SPAdes (Bankevich and Pevzner, 2016), Miniasm (Li, 2016), NextDenovo (https://github.com/Nextomics/NextDenovo), Smartdenovo (https://github.com/ruanjue/Smartdenovo), Flye (Kolmogorov et al., 2019) and Shasta toolkit (Shafin et al., 2020), that can produce good quality genomes which makes it difficult to choose which assembler to use. These assemblers, mainly based on Over-Layout-Consensus and De-Bruijn Graph algorithms, were developed to assemble genomes from human, plant, animal or bacteria. Briefly, Canu, wtdbg2, Miniasm and Smartdenovo are based on the overlap-Layout-Consensus algorithms, while Flye is based on a generalized Bruijn Graph. NECAT relays a novel progressive two-step error correction algorithm called NECAT with adaptive candidate-read selection for Nanopore raw reads (Chen et al., 2021). NextDenovo is a string graph-based *de novo* assembler for long reads. To date, there is limited available information on how to select *de novo* assembly tools or guidelines regarding how to evaluate the quality of an assembled genome using ONT data. In 2016, Sovic et al. (2016) compared the performance of five assembly tools for assembling ONT long reads from *E. coli* K-12 MG1655 and developed a framework for bacterial genome assembly. Several *de novo* assemblers were also evaluated these application in prokaryote whole genome assembly (Wick and Holt, 2019). Hyungtaek et al. (Jung et al., 2020) investigated the performance of five *de novo* tools in long reads from PacBio in 2020. Although they evaluated several criteria, such as CPU time, memory usage, contig numbers, N50 length and assembly accuracy, further investigation should be conducted to determine the performance of newly-developed assemblers, such as NextDenovo and NECAT. Whether these assemblers present a similar performance in Piroplasm genome assembly is a question that still needs to be investigated in the near future.

Babesiosis caused by pathogens of the genus *Babesia*, including economic and public health important species (*Babesia divergens*, *B. microti*, *B. crassa*, *B. motasi*, *B. bovis*), is one of the emerging and re-emerging tick-borne disease in the tropical and subtropical regions of the world. Till now, more than 100 *Babesia* species have been documented in human, wild and domestic animals. However, limited genomic information is available, which is one of main hinder to understand phylogenetic relationship, reveal gene family that may be critical for interactions between parasite and hosts or vectors. Genome size and GC content of these species ranges from ~6 Mbp to ~14 Mbp and ~36% to 50.6%. The possible reasons for low completeness of these genome is NGS short reads used to assemble genome, and even PacBio reads could not generate contiguity genome in some species, such as *Babesia divergens* and *B. ovata* (Guan et al., 2016; Yamagishi et al., 2017; Gonzalez et al., 2019). In other words, it is urgent to develop a high performance procedure of genome assembly for these species. In this study, we systematically evaluated publicly-available *de novo* assemblers in an attempt to provide answers to the following questions: 1) Which assembler will generate the ideal output? 2) What parameters should be applied to particular assembly tools and how they vary between organisms of the phylum? 3) What parameters should be used to evaluate the quality of a genome? 4) Is assembly correction using nanopore reads and Illumina seq-data required, and to what extent does merging individual and multiple assembly improve genomic quality? 5) What is the ideal output? Our results provided precise information that we then used for genome assembly using each assembler, which will make it possible for other researchers to replicate our work.

## Methods and Materials

### Sequencing and Preparation of Data

Two 6-month-old sheep were purchased from Jingtai county, Gansu Province, China, and confirmed to be free of piroplasm infection by microscopy, real time-PCR, nested PCR and ELISA assay (Guan G. et al., 2012; Yang et al., 2014; Niu et al., 2016; Yang et al., 2016). They were inoculated intravenously with 10 mL of cryopreserved blood infected with *B. motasi* Hebei. When parasitemia reached 20–40%, blood samples were collected into EDTA-coated tubes. Merozoites were purified from blood as previously described (Guan G. Q. et al., 2012). Genomic DNA was extracted using a commercial DNA extractions kit according to the manufacturer’s instructions (QIAamp DNA Blood Mini Kit; Qiagen, Hilden, Germany). The library for PromethION was constructed using a ligation kit (SQK- LSK109, Oxford Nanopore Technology, Oxford, UK) and then analyzed using two FLOMIN106 flow cells (v9.4.1). The raw FAST5 data were basecalled using Guppy (v3.2.2). The dataset was subsampled to six different coverages (approximately 15×, 30×, 50×, 70×, 100×, 120×) to test the effect of varying coverage on assembly quality. A library of 400-bp paired-end reads was sequenced using MGISEQ-2000RS (MGI Tech, Shenzhen, China). All test datasets are described in **Table 1**.

**Table 1:** Basic information of the datasets used for evaluation.

Accession number	Dataset	Description
CRA003898 (https://bigd.big.ac.cn/)	15X	reads from nanopore sequencing subsampled to coverage depth 15x, 18200 reads
	30X	reads from nanopore sequencing subsampled to coverage depth 30x, 37500 reads
	50X	reads from nanopore sequencing subsampled to coverage depth 50x, 56423 reads
	70X	reads from nanopore sequencing subsampled to coverage depth 70x, 77683 reads
	100X	reads from nanopore sequencing subsampled to coverage depth 100x, 117653 reads
	120X	reads from nanopore sequencing subsampled to coverage depth 120x, 144234 reads
CRA003907 (https://bigd.big.ac.cn/)	NGS reads	NGS reads used by assembly and base correction

For the Nanopore sequencing data, low-quality reads and contaminant reads were filtered by NanoFilt and NanoLyse (De Coster et al., 2018), respectively. Meanwhile, for NGS data, low quality base/reads and adaptor sequences were removed by trim_galore (https://github.com/FelixKrueger/TrimGalore).

### *De Novo* Assembly Tools and Assessment

We selected nine *de novo* genome assemblers-NECAT (v0.0.1), Canu (v2.2.2), wtdbg2 (v2.5), SPAdes (v3.15.2), Miniasm (v0.3), NextDenovo (v2.4.0), Smartdenovo, Flye (v2.8.3) and Shasta toolkit (v0.7.0), which are freely available and suitable for sequence assembling of long reads generated by the nanopore sequencing platform. For each depth of coverage, each assembly tool was run with different parameters until we achieved optimal results. Contig N50 was used as the primary metric to determine whether the assembler was suitable for assembly, as the largest contigs were usually preferred.

Reads correction is an important step in genome assembly, frequently taking much longer than the assembly itself. To carry out a fair comparison, the following pipeline was applied to this study: 1) After genome assembly from nanopore data with several depths of coverage, we employed minimap2 + Racon and Medaka to perform self correction using clear ONT data. 2) To evaluate whether the further error correction is essential using Illumina data, secondary correction using NextPolish was performed to generate the final assembly output (Hu et al., 2020). 3) We also merged assembly outputs derived from distinct *de novo* tools to generate more contiguous assembly.

Benchmarking Universal Single-copy Orthologs (BUSCO v5.1.3) was applied to determine the completeness of the genome assembly using the core apicomplexan dataset (apicomplexa_odb10). Then, to evaluate the sequence accuracy, alignment between genome assembly and Illumina-seq reads was performed using BWA (Li and Durbin, 2009). In addition, Samtools was employed to determine the reads coverage of the genome assembly.

## Results

### N50 Length and Contig Numbers Are Closely Related to Coverage Depth

**Figure 1:**
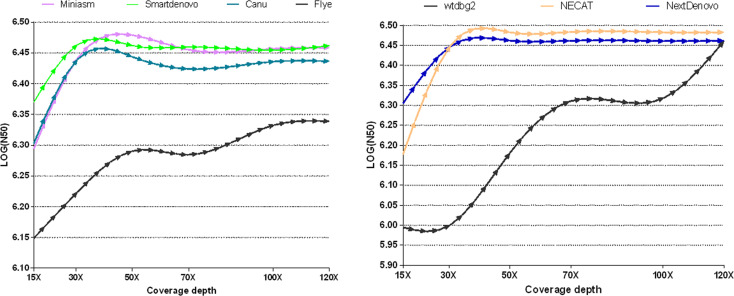
Comparison of the effect of various sequencing depths and assemblers on N50 length in *B. motasi* assemblies.

To measure whether and how the depth of coverage was related to the assembly performance of these nine tools, these freely-available tools were employed to assemble nanopore reads derived from *B. motasi* at different coverage depths (15×, 30×, 50×, 70×, 100×, 120×). It is clear from **Figure 1** that seven *de novo* tools showed an increase in performance with increasing coverage depth in respect of N50. The values of N50 lengths presented an upward trend with increasing coverage depth from 15× to approximately 40×. When the coverage depth exceeded 40×, very similar values of N50 length reached plateaus for NECAT, NextDenovo Smartdenovo and Miniasm, which were significantly greater than those of wtdbg2 and Flye. To be precise, NECAT achieved the greatest N50 length, while that of NextDenovo, Smartdenovo and Miniasm were comparable to each other for tested datasets and slightly lower than NECAT. Meanwhile, Canu achieved a moderately high level of N50 length (slightly lower than NextDenovo and Smartdenovo). With regard to low sequencing coverage depth, it was impossible to obtain an ideal N50 length for wtdbg2 compared to NECAT, Canu, NextDenovo, Smartdenovo and Miniasm. The N50 length of Flye showed an increase with increasing coverage depth; however the greatest value was still significantly lower than other *de novo* tools. When the depth coverage increased up to 120×, the lowest was observed in SPAdes with 464,701 base pairs ((LOG(N50) = 5.667)) and Shasta toolkit with 989,623 base pairs ((LOG(N50) = 5.995)), respectively.

The contig number was differentially affected by coverage depth. NECAT, NextDenovo, Flye and Miniasm reflected only slight changes with increasing depth of coverage, while for wtdbg2, Canu and Smartdenovo the numbers showed great changes (**Supplementary Figure 1** and **Supplementary Table 1**). The greatest contig number (>1200) was observed in SPAdes, even when coverage depth reached up to 120×. The second greatest contig number (198) was produced by Shasta toolkit. With respect to N50 and contig numbers, we excluded SPAdes and Shasta toolkit in our subsequent study making a comparison with other *de novo* assemblers.

### Computing Demand

Considering the computing environment and ease of operation, all tested tools are relatively user-friendly. All script and commands are provided in **Supplementary Information**. Computational demands are also important when selecting tools for *de novo* assembly. If tools require a lot of execution time and a great deal of memory usage, their use could be seriously limited. To reach a reasonable conclusion, we assigned 16 threads to genome assembly for the seven tools. Then, we measured the runtime demand for all seven tools (**Table 2**). This demonstrated that Miniasm was the fastest of all tested *de novo* assemblers, whereas Canu demanded the longest runtime for computation to assemble each coverage depth of dataset, compared with the other tools tested. According to the runtime, the seven tools could be classified as fast (Miniasm, wtdbg2 and NextDenovo), medium (Flye, Smartdenovo and NECAT) and slow (Canu) *de novo* assembly tools with increasing depth of coverage.

**Table 2:** Comparison of runtime in computational test using different coverage depth datasets.

	Depth of coverage					
	15ⅹ	30ⅹ	50ⅹ	70ⅹ	100ⅹ	120ⅹ
Run time (s)						
Miniasm	15	34	60	94	160	168
NextDenovo	123	210	351	360	391	464
Smartdenovo	280	733	1502	2472	4986	6764
wtdbg2	382	524	662	667	811	945
Flye	836	1498	2035	3022	4380	7619
NECAT	1374	2562	3261	4311	4963	5940
Canu	22020	25740	49440	80220	116761	128097

### Genome Completeness

**Figure 2:**
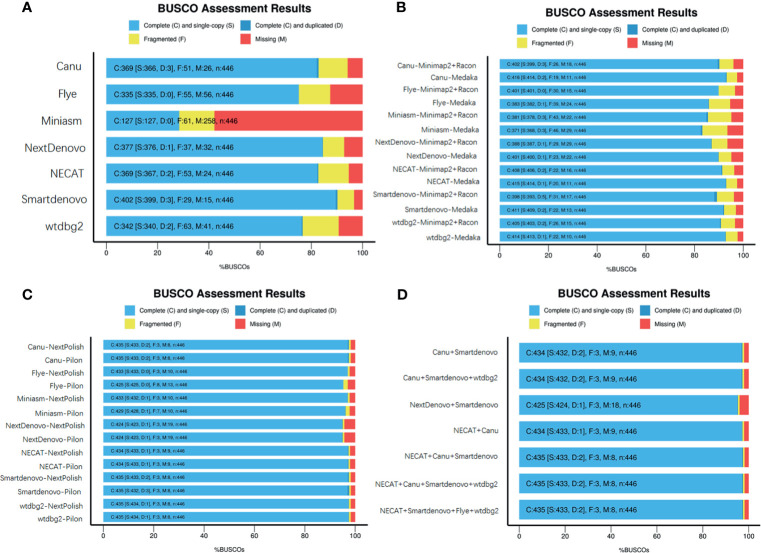
Assessment of genome assemblies using BUSCO. **(A)** Outputs from assemblies without further base correction; **(B)** Assemblies corrected using Minimap2 + Racon or Medaka with ONT reads; **(C)** Assemblies corrected using NextPolish or Pilon with Illumina reads; **(D)** Merged multiple assemblies corrected with nanopore and NGS reads.

These seven *de novo* tools were employed to generate long contigs from nanopore sequencing long reads. Assessments of genome completeness of *de novo* tools showed that Miniasm yielded the lowest values, while relatively low values were observed in Miniasm with completeness less than 30%. Relatively high levels of genome completeness were observed in Canu, Flye, NECAT, NextDenovo and Smartdenovo (**Figure 2A**).

To determine whether base correction is capable of improving the completeness of assembled genomes, two steps of genome polishing were employed using nanopore data and NGS reads. First, minimap2 + Racon and Medaka (https://github.com/nanoporetech/medaka) were used to perform correction using ONT long reads. This correction step greatly facilitated the completeness of the genome for all *de novo* assemblies (**Figure 2B**). Medaka requires less runtime than minimap2 + Racon and contributes better to improve the completeness of genomes. Particularly in Miniasm, Flye and wtdbg2, the figures increased from 28.5 to 85.5%, 75.1 to 85.9% and 76.6% to 92.8%, respectively, while for other assembly tools, there were slight increases in genome completeness. Considering the high error rate of ONT reads, the second correction step was performed using NGS reads with the nextPolish and Pilon software package (Walker et al., 2014). As expected, the figures for all seven *de novo* tools increased from ~85% to ~95%. As an alternative, we assessed the genome completeness of the correctional genome, in which the second correction step was directly performed against the assembled outputs generated from these *de novo* tools, and found that it could also achieve a level almost equal to the two steps of genome polishing. NextPolish and Pilon showed a similar performance in improving the genome completeness (**Figure 2C**).

We next measured the percentage of genome coverage by calculating the assembly aligned to the Illuminia short reads (**Table 3**). Although assembly quality for Flye and wtdbg2 presented a noticeable low in terms of N50 and contig numbers, these genomes managed to cover almost the whole genome (>95%). Relatively high genome coverage was also achieved by NECAT, Canu and Smartdenovo. It seems that more than 30× coverage was sufficient for NECAT, Canu and NextDenovo to generate good outputs. In addition, the Samtools software package was applied to evaluate the accuracy of assemblies by calculating single nucleotide polymorphisms (SNPs). The values of SNPs were obtained by aligning NGS reads with each assembly (**Supplementary Table 1**). Most accurate assemblies were generated by NECAT and Smartdenovo, while Flye produced low quality assembly.

**Table 3:** Genome coverage percentages of assemblies from different sequencing depths.

	Depth of coverage					
	15ⅹ	30ⅹ	50ⅹ	70ⅹ	100ⅹ	120ⅹ
Genome coverage (%)						
NextDenovo	97.62	98.23	98.36	98.01	97.86	97.91
Smartdenovo	97.54	98.54	98.62	98.54	98.58	98.75
wtdbg2	96.63	92.31	91.33	97.62	96.94	97.13
Flye	95.12	95.58	94.81	95.83	95.23	96.05
NECAT	98.01	98.64	98.41	98.4	98.36	98.59
Canu	97.78	98.50	98.78	98.82	98.57	98.81
Miniasm	94.81	95.26	95.83	95.37	95.46	95.91

### Comparison of Seven *De Novo* Assemblers Using Multiple Parameters

**Figure 3:**
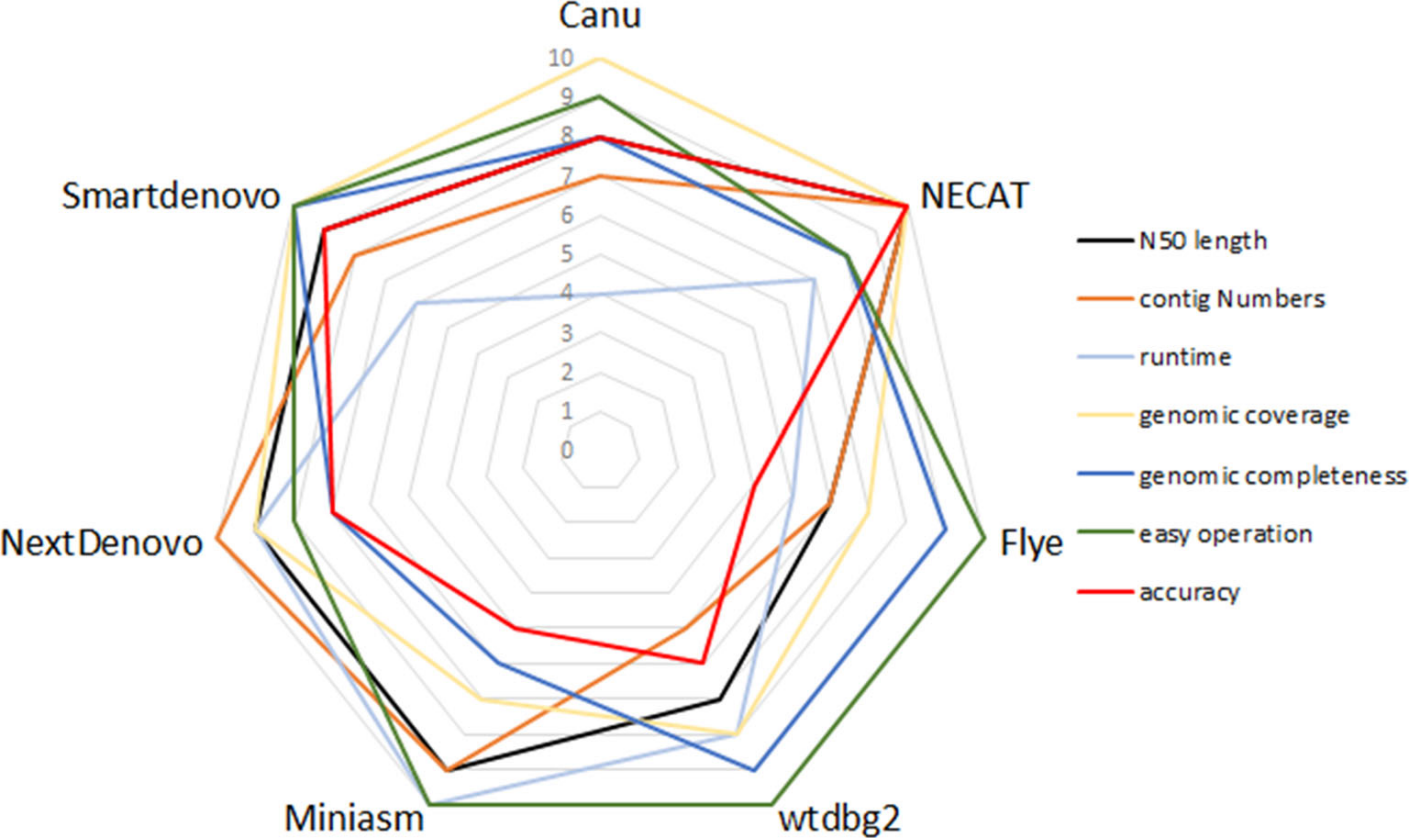
Relative performance of *de novo* assemblers in terms of quality of genome and recommendation.

To compare the performance of these *de novo* tools, each assembler was given a score ranging from 4 to 10 in respect of runtime, N50 length, contig numbers, computation demand, ease of operation, genomic coverage and genomic completeness. To be exact, each assembler was ranked from best to worst for specific criteria, so the best performance of the tool was scored as 10, whereas the worst was given a score of 4. **Figure 3** provides a summary of the advantages and disadvantages of these seven tested assemblers.

### Genome Post-Processing After Assembling

To evaluate whether merging genomes from each assembler could yield a high quality genome, quickmerge was employed to generate a merged assembly (https://github.com/mahulchak/quickmerge). As the quality of the input assembly directly affects that of the final output, we used corrected assembly using ONT reads and Illumina seq-data as inputs to evaluate post-processing assembly performance. We tested all 120 possible combinations, from merging two individual assemblies to seven assemblies, and the results indicated that some merging improved completeness of the genome, contig size and genome contiguity. The top eight combinations of individual and multiple inputs are listed in **Figure 2D** and **Supplementary Table 1**, which had positive effects on improving the completeness of the genome, resulted in longer contigs and a less contig numbers (**Figure 2D** and **Supplementary Table 1**).

## Conclusions and Discussions

N50 length has been widely used as a metric of assembly contiguity. However N50 is not always a “gold standard” to assess the performance of assemblers (Yandell and Ence, 2012). It is critical to note that a larger N50 is not always reasonable when long reads are not correctly connected (Salzberg et al., 2012). These assemblies can generate large contigs, but result in worse assembly. In an extreme case, an assembly with the largest N50 could contain a very long scaffold and many short scaffolds (Bradnam et al., 2013). Because a reference genome was not available in this study, assembly accuracy could not be evaluated by aligning to the reference genome. When the genome assembly is finished, the main purpose of a genome project is to denote the gene structure and function. Consequently gene completeness is an alternative metric. The results of evaluation of genome completeness using Benchmarking Universal Single-copy Orthologs (BUSCO) and genome coverage revealed that high quality assembly was achieved. Yandell et al. (Yandell and Ence, 2012) proposed that a “gene sized” scaffold N50 could be a preferable criterion. A good assembly has the largest number of scaffolds that are greater than the “gene sized” scaffold N50. In our assembly, all contigs generated from the seven tested *de novo* tools were longer than the mean length of the gene in Apicomplexa (Gardner et al., 2005; Cornillot et al., 2012; Yamagishi et al., 2017; Bogema et al., 2018).

Alignment reads to assembly can be used to assess the assembly quality in terms of the completeness and accuracy, which are critically important for multiple applications in subsequent studies. Transcriptome data and genome sequencing data could be mapped back to assembly to evaluate the quality of the genome assembled. As transcriptome data were not available in this study, we focused our attention on mapping the Illumina reads to assembled contigs. It is also considered as one of the efficiency criteria of the assembler. Commonly, a good-performing *de novo* tool has a high genome coverage. Except for SPAdes, good genome coverage was produced by all tested assemblers, reflecting a stable performance among the different datasets. According to this metric, when the coverage depth was over 30×, NECAT, Canu, NextDenovo and Smartdenovo yielded admirable results with ~98% of genome coverage. However Miniasm, Flye and wtdbg2 were found not to be good choices as candidate assemblers.

As expected, error correction procedure can increase both contiguity of assembly and completeness of corn conserved genes. It was noticed that except for Smartdenovo, assemblers (Miniasm, wtdbg2 and Flye) without an included error correction module generated less contiguity assembly or low level completeness of the genome, whereas NECAT, Canu and NextDenovo presented good performance in respect of N50 and BUSCO assessment. Further base corrections with nanopore data and Illumina data had greater or lesser positive effects on the quality of assembly, which could be observed in the results of BUSCO assessments.

Furthermore, post-processing assembly, such as merging different assemblies from several assemblers, was performed to create conserved genome regions to reduce the complexity of *de novo* assembly. We tested 120 possible combinations, which merged multiple assemblies, generated from seven *de novo* tools, to assess their contribution to improving the quality of the genome. Based on our observation, although the outputs of post-processing assembly neither contributed to contig size nor to BUSCO assessment results, several merges improved these of the assembly, such as Canu + NECAT, Canu + Smartdenovo, and NextDenovo + Smartdenovo. Hyungtaek et al. and Alhakami et al. proposed that increasing the number of inputs provided a great contribution to improving the contiguity (more longer N50) of assembly; however, a similar situation was not observed in our study. This may vary between different datasets and different genome structures.

In this study, limited datasets were used to evaluate the performance of nine *de novo* assemblers, so to identify whether the optimized pipelines of *de novo* assembly present similar performance in other species will need to be investigated in the near future. Any of Canu, Flye or Miniasm + minimap + Racon presented a good performance in genome assemblies of plants and crops (Jung et al., 2020). In contrast, when post-processing assembly was not performed, neither Flye nor Miniasm proved a good choice for genome assembly in the present study. Again for SPAdes, it has advantages in the hybrid assembly pipeline, which uses ONT reads and NGS reads as inputs (Sovic et al., 2016). In addition, Shasta toolkit enables efficient *de novo* assembly of the human genome in terms of accuracy, speed and contiguity (Shafin et al., 2020). Conversely, SPAdes and Shasta toolkit produce less contiguity of assemblies in *B. motasi*. Taking the available results together, a benchmarking framework for *de novo* assembly may present different performances among various organisms. That is to say, high performance of an assembly framework should be developed targeted to a specific species. Our results provided a reference, when weighing the advantages and disadvantages of each tested *de novo* tool, for selecting acceptable tools, designing sequencing projects, and improving the quality of assemblies (**Figure 3**).

**Figure 4:**
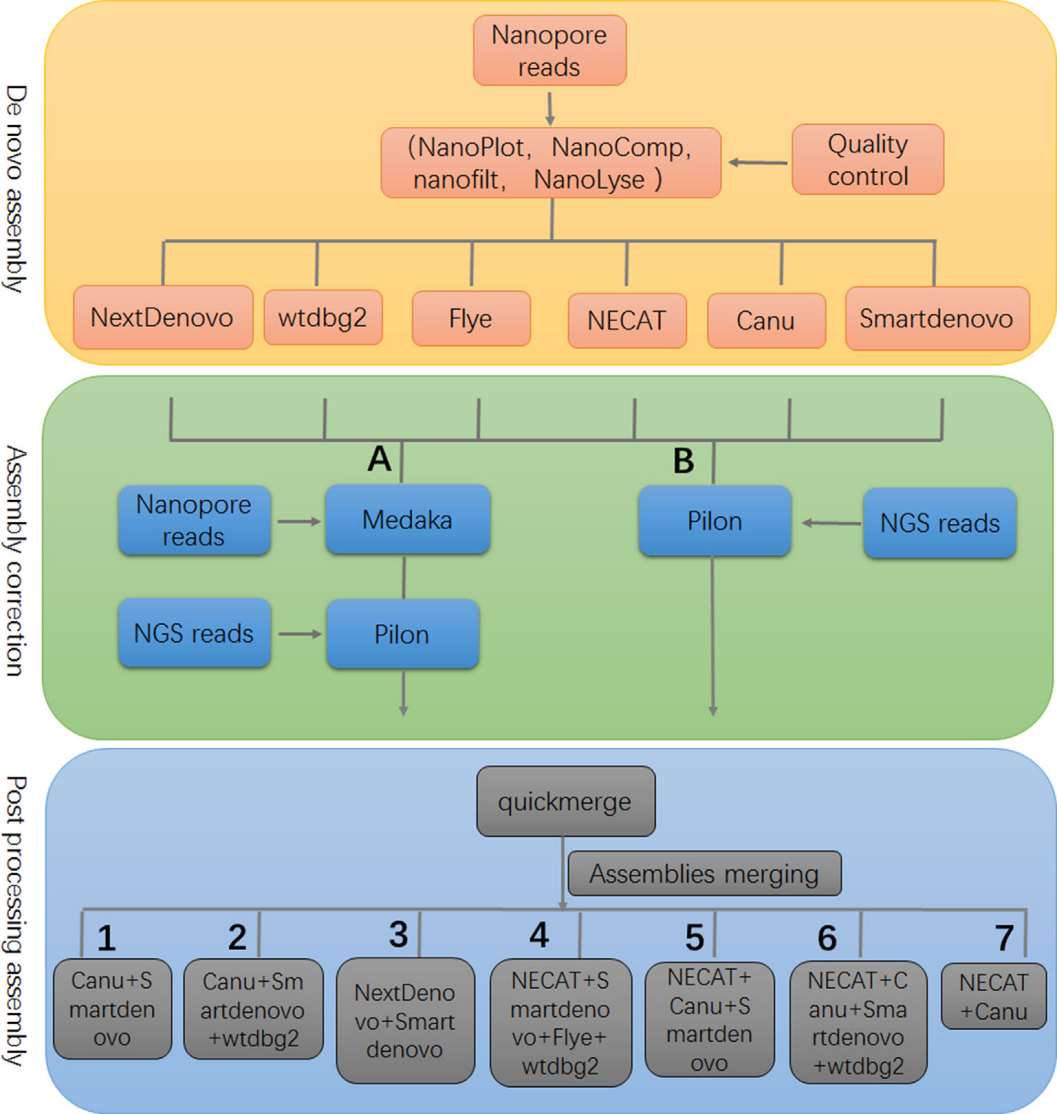
The framework of genome assembly. In the stage of assembly correction, either strategy **(A)** or **(B)** could achieve similar results. There were seven optional post-processing strategies that are recommended to produce a high quality assembly.

A limitation of our study was that relatively small datasets were used to evaluate the performance of assemblers; however, the genome assembly framework, developed in this study, provided valuable information for genome analyses of piroplasm parasites (**Figure 4**). Determining and understanding the limitations of specific tools may provide critical information for *de novo* genome assembly and point the direction for improving performance of current tools and developing highly-efficient assemblers.

## Data Availability Statement

The data presented in this study are deposited in China National Center for Bioinformation and accession numbers are CRA003898 and CRA003907 (https://bigd.big.ac.cn/).

## Ethics Statement

The collection and manipulation of sheep blood samples was approved by the Animal Ethics Committee of the Lanzhou Veterinary Research Institute, Chinese Academy of Agricultural Sciences. All sampling procedures were handled in accordance with the Animal Ethics Procedures and Guidelines of the People’s Republic of China (Permit No. LVRIAEC-2018-001). All the procedures conducted were according to the Ethical Procedures and Guidelines of the People’s Republic of China.

## Author Contributions

Manuscript, JW. Reagents/materials/analysis, JW, KC, QR, JX, JLL, GW, AL, YL, and YZ. Supervision, GL, JXL, WM, HY, and GG. All authors contributed to the article and approved the submitted version.

## Funding

This study was financially supported by the National Key Research and Development Program of China (2017YFD0501200; 2018YFD050230; 2017YFD0500904; 2017YFD0502300), the 973 Program (2015CB150300), ASTIP (CAAS-ASTIP-2016-LVRI), NBCIS (CARS-37), the Jiangsu Co-innovation Center program for Prevention and Control of Important Animal Infectious Diseases and Zoonoses, the Protist 10,000 Genomes Project (P10K) Consortium.

## Conflict of Interest

Author YZ was employed by the company Lanzhou Baiyuan Gene Technology Co., Ltd.

The remaining authors declare that the research was conducted in the absence of any commercial or financial relationships that could be construed as a potential conflict of interest.

## Publisher’s Note

All claims expressed in this article are solely those of the authors and do not necessarily represent those of their affiliated organizations, or those of the publisher, the editors and the reviewers. Any product that may be evaluated in this article, or claim that may be made by its manufacturer, is not guaranteed or endorsed by the publisher.

## Supplementary Material

Supplementary Figure 1 Comparison of the effect of various sequencing depths and assemblers on contig numbers in *B. motasi* assemblies.
