# Supplementary figures and images for: Systematic Comparison of the Performances of De Novo Genome Assemblers for Oxford Nanopore Technology Reads From Piroplasm
Source: Front Cell Infect Microbiol. 2021 Aug 16;11:696669. doi: 10.3389/fcimb.2021.696669 (PMC8415751; doi:10.3389/fcimb.2021.696669)

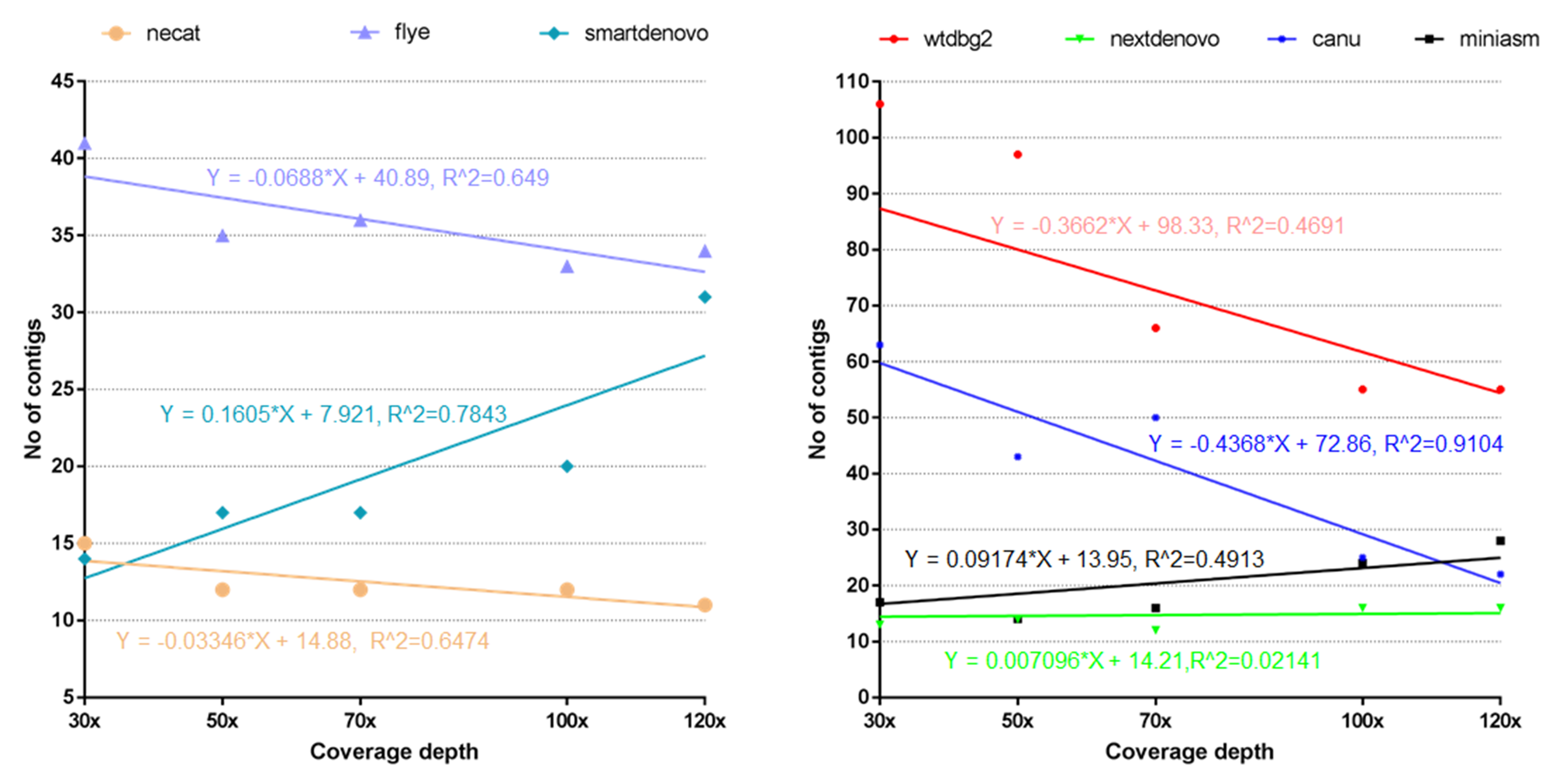

Supplement: Supplementary Figure 1 — Comparison of the effect of various sequencing depths and assemblers on contig numbers in B. motasi assemblies. [file Image_1.tif]
