# Supplementary material for: Systematic Comparison of the Performances of De Novo Genome Assemblers for Oxford Nanopore Technology Reads From Piroplasm
Source: Front Cell Infect Microbiol. 2021 Aug 16;11:696669. doi: 10.3389/fcimb.2021.696669 (PMC8415751; doi:10.3389/fcimb.2021.696669)
Supplement: Supplementary file 2 [file Table_1.docx]

| Assemblers | contigs (>= 25000 bp) | contigs (>= 50000 bp) | contigs | Total length (>= 25000 bp) | Total length (>= 50000 bp) | Largest contig | Total length | GC (%) | N50 | SNP |
| --- | --- | --- | --- | --- | --- | --- | --- | --- | --- | --- |
| Canu | 22 | 22 | 22 | 14706224 | 14706224 | 3316906 | 14706224 | 46.84 | 2740159 | 2204 |
| Flye | 27 | 23 | 29 | 13532539 | 13387700 | 3000448 | 13558922 | 46.86 | 2166847 | 12118 |
| NECAT | 11 | 11 | 11 | 14591341 | 14591341 | 4497234 | 14591341 | 46.94 | 3041312 | 1375 |
| nd | 14 | 14 | 14 | 14452201 | 14452201 | 3025618 | 14452201 | 46.88 | 2884564 | 3730 |
| sd | 31 | 22 | 31 | 15804932 | 15436617 | 3186500 | 15804932 | 46.83 | 2900192 | 1544 |
| wtdbg | 29 | 18 | 48 | 14679279 | 14316296 | 3050828 | 14968894 | 46.8 | 2899535 | 4186 |
| Miniasm | 25 | 15 | 28 | 15395347 | 15099229 | 4621787 | 15421473 | 46.56 | 2873366 | 8879 |
| SPAdes | 49 | 31 | 1435 | 10530535 | 9953358 | 1291895 | 15594837 | 46.13 | 255398 | - |
| Shasta toolkit | 56 | 32 | 198 | 13458880 | 12623395 | 2014803 | 14218783 | 46.83 | 989623 | - |
| Canu+NECAT | 12 | 12 | 12 | 14786703 | 14786703 | 4510228 | 14786703 | 46.84 | 3044224 | - |
| Canu+sd | 11 | 11 | 11 | 14675773 | 14675773 | 4527126 | 14675773 | 46.87 | 3045872 | - |
| NECAT+sd | 10 | 10 | 10 | 14560120 | 14560120 | 4516999 | 14560120 | 46.85 | 3045872 | - |
| Canu+sd+wt | 11 | 11 | 11 | 14686034 | 14686034 | 4527126 | 14686034 | 46.87 | 3045872 | - |
| NECAT+Canu+sd | 11 | 11 | 11 | 14664118 | 14664118 | 4497234 | 14664118 | 46.94 | 3044876 | - |
| NECAT+Canu+sd+wt | 11 | 11 | 11 | 14762187 | 14762187 | 4497234 | 14762187 | 46.95 | 3045872 | - |
| NECAT+sd+Flye+wt | 12 | 12 | 13 | 14858545 | 14858545 | 4497234 | 14864552 | 46.85 | 3076762 | - |

Supplement Table 1. Statistical information of assemblies generated by different assemblers

sd: Smartdenovo; nd: NextDenovo; wtdbg2: wt
